# Measuring Spinal Mobility Using an Inertial Measurement Unit System: A Reliability Study in Axial Spondyloarthritis

**DOI:** 10.3390/diagnostics11030490

**Published:** 2021-03-10

**Authors:** Megan O’Grady, Tom O’Dwyer, James Connolly, Joan Condell, Karla Muñoz Esquivel, Finbar D. O’Shea, Philip Gardiner, Fiona Wilson

**Affiliations:** 1 Discipline of Physiotherapy, Trinity College Dublin, D08 W9RT Dublin, Ireland; odwyertk@tcd.ie (T.O.); wilsonf@tcd.ie (F.W.); 2 Independent Researcher, D08 W9RT Dublin, Ireland; 3 Letterkenny Institute of Technology, Letterkenny, F92 FC93 Donegal, Ireland; james.connolly@lyit.ie; 4 Magee Campus, Intelligent Systems Research Centre, Faculty of Computing, Engineering and the Built Environment, Ulster University, Derry/Londonderry BT48 7JL, UK; j.condell@ulster.ac.uk (J.C.); kc.munoz-esquivel@ulster.ac.uk (K.M.E.); 5 Rheumatology Department, St James’s Hospital, D08 NHY1 Dublin, Ireland; FOShea@stjames.ie; 6 Western Health and Social Care Trust, Londonderry BT47 6SB, UK; Philip.Gardiner@westerntrust.hscni.net

**Keywords:** axial spondyloarthritis, spinal mobility, inertial measurement unit, reliability

## Abstract

The objectives of this study were to evaluate the reliability of wearable inertial motion unit (IMU) sensors in measuring spinal range of motion under supervised and unsupervised conditions in both laboratory and ambulatory settings. A secondary aim of the study was to evaluate the reliability of composite IMU metrology scores (IMU-ASMI (Amb)). Forty people with axSpA participated in this clinical measurement study. Participant spinal mobility was assessed by conventional metrology (Bath Ankylosing Spondylitis Metrology Index, linear version—BASMI_Lin_) and by a wireless IMU sensor-based system which measured lumbar flexion-extension, lateral flexion and rotation. Each sensor-based movement test was converted to a normalized index and used to calculate IMU-ASMI (Amb) scores. Test-retest reliability was evaluated using intra-class correlation coefficients (ICC). There was good to excellent agreement for all spinal range of movements (ICC > 0.85) and IMU-ASMI (Amb) scores (ICC > 0.87) across all conditions. Correlations between IMU-ASMI (Amb) scores and conventional metrology were strong (Pearson correlation ≥ 0.85). An IMU sensor-based system is a reliable way of measuring spinal lumbar mobility in axSpA under supervised and unsupervised conditions. While not a replacement for established clinical measures, composite IMU-ASMI (Amb) scores may be reliably used as a proxy measure of spinal mobility.

## 1. Introduction

Axial spondyloarthritis (axSpA) is a complex chronic inflammatory disease predominantly affecting the axial skeleton [1]. In the early stages of the disease, restriction in spinal mobility is mainly due to reversible inflammation in and around the spine, but in later stages the restriction becomes permanent due to structural bony damage [2,3]. Monitoring of individuals with axSpA should center on aspects of the disease that cause symptoms or functional disability [4], and which are subject to change as the disease progresses or treatment is introduced, such as decreased spinal mobility [5,6].

Spinal mobility has been recognized as an important outcome in the management of axSpA and has been included in the Assessment of Spondyloarthritis International Society (ASAS) core set for clinical assessment in axSpA [6,7]. The Bath Ankylosing Spondylitis Metrology Index (BASMI) is a well-established method of measuring spinal movement in axSpA [8]. While the BASMI is a low-cost tool with minimal training and equipment required, it cannot be performed independently, limiting its utility outside of the clinical setting. It also lacks the sensitivity to change required to monitor disease progression [9,10,11]. As a result of these concerns, the BASMI failed to achieve approval by the ASAS group as a core outcome measure in axSpA [5]. There is a rapidly growing role for telemedicine as a tool to improve care for individuals with rheumatic disease, although there is a recognition that limitations in technology need to be understood and addressed to achieve standards of care consistent with existing in-person services [12]. There is therefore a need for a reliable and sensitive measure of spinal mobility to be used in studies of drug and physical interventions in axSpA.

Video-based optoelectronic systems are often thought of as the laboratory gold standard for human motion analysis [13,14]. These systems can provide complex descriptions of body segment motion but only measure movement over a short period of time. Their capture area can be limited by cameras, body markers and (environmental) equipment positioning, and they create artificial environments for movement assessment. Due to the high cost of equipment and training, they are therefore not feasible for many research centers or real-world testing.

In recent years, wearable inertial motion unit (IMU) sensor systems have advanced to the point of offering a viable method of clinically measuring spinal mobility [13,14] and analyzing spinal posture [15]. An IMU sensor typically incorporates a tri-axial accelerometer, gyroscope and magnetometer, and several can be incorporated unobtrusively as part of a wearable sensor system. The validity and reliability of such systems in the measurement of lumbar spine mobility have been established in healthy populations [16,17]. The validity and reliability of an IMU sensor-based system for evaluating cervical and lumbar spinal mobility in individuals with axSpA were recently established under supervised conditions [18,19]. If the full range of spinal mobility can be reliably measured in unsupervised ambulatory settings using IMU sensor-based systems, this tool could provide a viable method of reducing variability in measurement of spinal mobility, be sensitive to small changes in mobility over time, and be an important step towards digital self-management. It could be used by individuals with axSpA and clinicians involved in their care to reliably monitor signs remotely, providing clinicians with a “real-life” assessment of current disease state.

This study is the third in a project [18,19] investigating wearable IMU sensors and composite metrology scores in individuals with axSpA, with a focus on reliability in the ambulatory setting. The primary aim of this study was to assess the reliability of spinal IMU sensors in measuring spinal mobility of individuals with axSpA. The objectives were to evaluate the reliability of spinal IMU sensors in measuring spinal range of motion (1) under supervised and unsupervised conditions in the exercise laboratory, and (2) under unsupervised conditions in an ambulatory setting. A secondary aim of the study was to evaluate the reliability of calculated composite IMU metrology scores (IMU-ASMI (Amb)) and to establish correlations with BASMI. The reader is advised to refer to Gardiner et al. [19], Aranda-Valera et al. [18], and to the Supplementary Materials for a detailed explanation of the IMU-ASMI (Amb) score.

## 2. Materials and Methods

### 2.1. Study Design

This was a clinical measurement study with a specific focus on reliability. The study was approved by the local research ethics committee [REC Reference: 2017-10 List 37 (20)].

### 2.2. Participant Eligibility and Recruitment

Inclusion criteria for the study were as follows: diagnosis of axSpA (by ASAS criteria) made at least six months prior to recruitment to the study, age between 18 years and 80 years old and the ability to read and understand the English language. Exclusion criteria were severe joint or spinal pain at the time of the study, prior total hip arthroplasty or severely restricted hip movement, history of previous vertebral fracture, history of previous spinal surgery, severe scoliosis, spinal deformity or complete segmental fusion of the spine, pregnancy or being unable to mobilize without assistance or mobility aid.

Participant selection was through convenience sampling. Potential participants attending a dedicated hospital-based axSpA clinic were informed of the study by a gatekeeper who was not part of the research team. Notice of the study was also circulated via the social media channels of the Ankylosing Spondylitis Association of Ireland and Arthritis Ireland, and sent to individuals who were on a register having expressed interest in taking part in research projects. Interested persons contacted the study team and were screened for eligibility over the phone or via email. Participant diagnosis was confirmed by letter from the participant’s rheumatologist or general practitioner.

### 2.3. Data Collection and Baseline Assessments

Socio-demographic (age, sex and employment status) and anthropometric (weight, height and BMI) data were collected. Condition-specific data (time since onset of symptoms, time since diagnosis, medications and HLA-B27 status) were self-reported by participants. A battery of clinical questionnaires were self-completed by participants. These were: the Bath Ankylosing Spondylitis Disease Activity Index (BASDAI) [20], the Bath Ankylosing Spondylitis Functional Index (BASFI) [21], and the Bath Ankylosing Spondylitis Global Score (BAS-G) [22].

**Figure 1:**
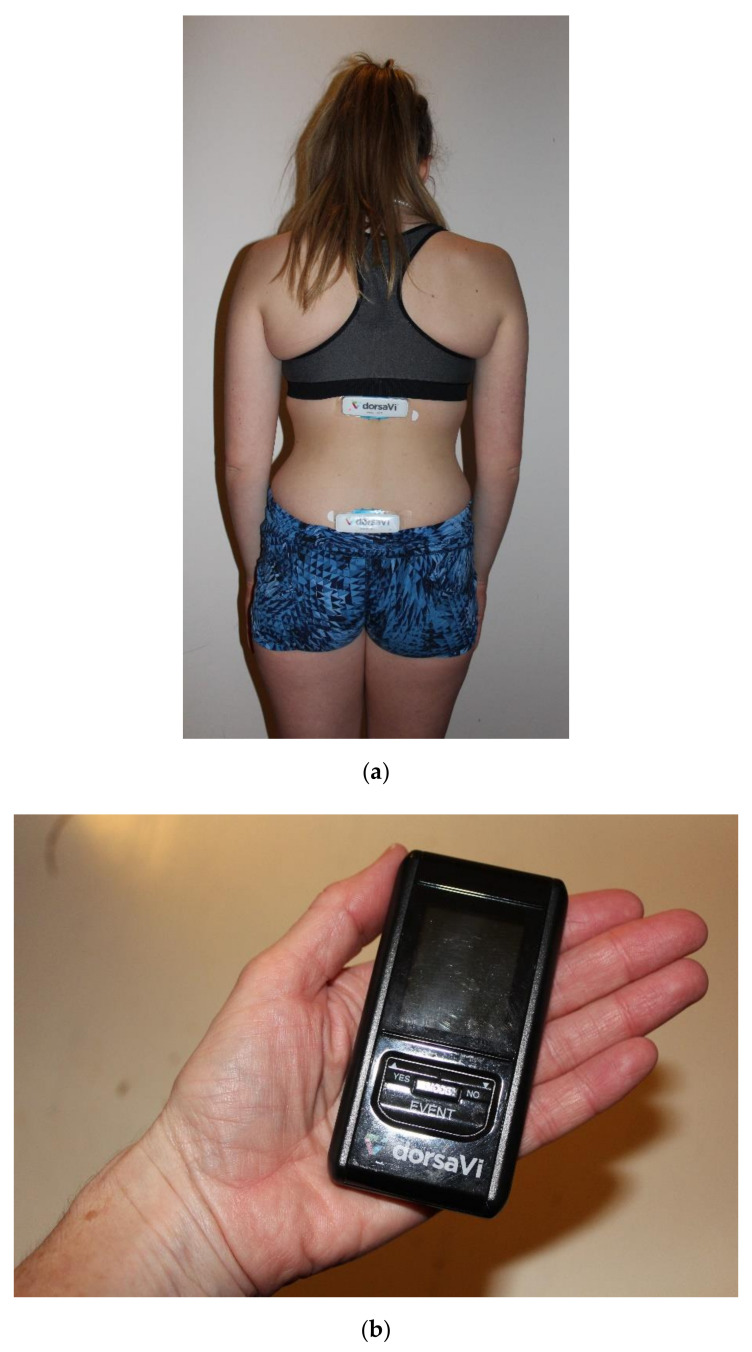
(**a**) ViMove^TM^ sensor location; (**b**) Pocket recording device used by participant.

The ViMove^TM^ wireless sensor kit (DorsaVi^TM^, Melbourne, VIC, Australia) was used as an IMU sensor-based system to measure spinal range of movement. Members of the research team attended a half-day training course to ensure that sensor application and movement tests were carried out according to the manufacturer’s standardized protocols. The ViMove^TM^ system uses two IMU sensors to provide an absolute orientation estimation (roll, pitch, and yaw) and calculate the relative orientation in three planes (sagittal, frontal and transverse) by combining the measurements of both sensors. The sensors connect and transmit IMU data using radio frequency to a pocket recording device at a frequency of 20 Hz, from which data can be downloaded or viewed directly from a laptop (see Figure 1). The ViMove^TM^ sensor setup was previously validated against both the Fastrak and Vicon motion sensor systems [17,23]. Aranda-Valera et al. [18] recently established the validity of the sensor setup in evaluating spinal mobility in individuals with axSpA using an optical motion capture system as a reference.

### 2.4. Assessment Schedule

Eligible participants attended the test center for assessment on two consecutive days. A research physiotherapist (MOG) trained in assessing individuals with axSpA carried out clinical tests. Both assessments were at approximately the same time each day. The phase between the two appointments in the laboratory was a community-based ambulatory phase, during which participants were unsupervised. Table 1 summarizes the testing schedule.

On Day 1, socio-demographic data were recorded and anthropometric measurements were completed. Following a five minute warm-up (treadmill walking or stationary exercise bike depending on participant preference), chest expansion and spinal mobility using the linear versions of the Bath Ankylosing Spondylitis Metrology Index (BASMI_Lin_) were recorded following ASAS guidelines [6,8,24].

The two sensors were then attached to the participant according to the manufacturer’s guidelines. The lower (sacral) sensor was positioned using a line drawn between the posterior superior iliac spines, and the upper (trunk) sensor was positioned above this line using DorsaVi^TM^ designed height-specific templates to ensure the accurate positioning of the upper sensor over the T12 vertebra (see Figure 1). Both sensors were mounted into a baseplate attached to an adhesive strip, which was placed directly on the skin. Calibration of the system was performed in relaxed standing (as per the manufacturer’s standardized protocol) and angles were recorded at the zero position for each IMU sensor to set the baseline position. Each sensor then calculated orientation angles with respect to this calibrated starting position.

Using standardized instructions, the assessor verbally guided the participants through a sequence of spinal movements: flexion, extension, lateral flexion (left then right), and rotation (left then right). Each movement was repeated three times before moving to the next movement (Condition 1: laboratory, supervised). Participants were then instructed to repeat the same sequence of spinal movements without supervision (Condition 2: laboratory, unsupervised). Participants followed either an instructional video (an example is included in the Supplementary Materials) or written instructions (depending on preference); the same standardized instructions were used as during the supervised tests. The assessor left the room until all movements in the sequence were completed. Participants were instructed to press an ‘event’ button on the wireless pocket recorder when they were about to begin each movement, and again when they had completed the movement.

Participants left the exercise laboratory with the two IMU sensors in situ. During this ambulatory phase, participants repeated the spinal movement sequence at home by following video or written instructions and pressing the ‘event’ button on the wireless recorder (Condition 3: Ambulatory, unsupervised). The following day, participants returned to the exercise laboratory. The BASMI_Lin_ and the same spinal movements were repeated under supervised and unsupervised conditions. As test sessions were at different times of day, the diurnal variation in symptoms was monitored by participants recording their levels of pain and fatigue on a numerical rating scale (Pain NRS from ‘0—No pain’ to ‘10—Most severe’ and Fatigue NRS from ‘0—None’ to ‘10—Very severe’) prior to and after completing the spinal movements [25].

### 2.5. Data Management

#### 2.5.1. Sensor Data Output

Data was downloaded from sensors after each phase of testing using Microsoft Excel for Windows version 2013 (Microsoft Corporation, Redmond, WA, USA). The start and end of movement tests were identified using ‘event’ markers, and minimum and maximum degrees of movement were generated within each set of event markers. The data analyst visually inspected each movement test, and adjusted the start and end of the movement window if needed, to ensure they coincided with actual spinal movement. Each movement was repeated three times, and the maximum degree of movement was computed from the available repetitions. The mean of these degree of movement values was used in subsequent calculations. Output for rotation movements was only available under supervised conditions owing to technical limitations with the system. Output was designated as Trunk (from the upper sensor, the orientation angle from the upper lumbar sensor to the ground; represents lumbar and pelvic movement) and Lumbar (the angle between the upper sensor and the sacral sensor; represents lumbar movement). The ‘full-arc’ range of movement for a given spinal movement test was calculated. The reliability of full-arc movements has been shown to be higher than measurements from midline [19].

Minimum, maximum and range data were independently validated by examining the raw IMU sensor data for each test. A random selection of *n* = 5 participant data samples (12.5% of all samples) were analysed using Microsoft Excel. The event markers corresponding with the start and end of each spinal movement test were again visually analysed by an independent reviewer, and Excel-generated data values for each movement were numerically compared with the corresponding values generated by the ViMove^TM^ software for each movement test. Results showed that there were no discrepancies between data generated by both methods for day 1 and day 2 of laboratory data. There was a comparison variation of 1.09 degrees within all ambulatory data samples. This was considered an acceptable amount of variation.

#### 2.5.2. Calculation of Composite Metrology Score—IMU-ASMI (Amb)

Normalized scales permit rapid evaluation of mobility, without the need for clinicians to know normal ranges of movement. Each sensor-based movement test (Flexion-Extension, Lateral Flexion L + R, and Rotation L + R) was converted into a normalized index using a formula based on that used to calculate BASMI_Lin_ [24]. The 10th and 90th percentile ranges for each sensor-based movement test were obtained from research cohorts associated with this research group (Cordoba healthy controls, Altnagelvin AxSpA cohort). Normalized scores were calculated as follows:$\left(\left(90\mathrm{th}\;\mathrm{centile}\;-\;A\right)/\left(90\mathrm{th}\;\mathrm{centile}\;-\;10\mathrm{th}\;\mathrm{centile}\right)\right)/10)$; A = range of motion in degrees). If A ≥ 90th centile, the normalized score = 0; if A ≤ 10th centile, the normalized score = 10. Composite IMU-ASMI (Amb) scores were calculated for the lumbopelvic region (Trunk-ASMI) and the lumbar region (Lumbar-ASMI). Trunk-ASMI and Lumbar-ASMI were calculated as the mean of the normalized scores of the lumbopelvic region and lumbar region, respectively. The reliability of regional composite indices has been shown to be superior to that of individual components [19]. The reader is advised to refer to the Supplementary Materials for a detailed explanation of the IMU-ASMI composite metrology score.

### 2.6. Statistical Methods

Descriptive data are presented as frequencies and percentages for categorical variables, and continuous data were presented as mean and standard deviation, or median and interquartile range, as appropriate.

Test-retest reliability, compared across laboratory conditions (supervised and unsupervised) and ambulatory conditions, was evaluated using intra-class correlation coefficient (ICC) and standard error of measurement (SEM). Two-way, mixed-effects, single rater, absolute agreement model for ICCs were used. ICC interpretation was as follows: <0.5 = poor, 0.5 to 0.75 = moderate, 0.75 and 0.9 = good, >0.90 = excellent [26]. The SEM was calculated as follows: SEM = SD × √(1-ICC), with SD representing the pooled (two measurements) SD of the measure. Agreement between movement tests under each condition was evaluated using Bland-Altman analysis. The mean bias and the limits of agreement (LoA) were calculated to provide an estimate of the interval in which 95% of the differences between both test conditions are.

Correlations between BASMI_Lin_ and IMU-ASMI (Amb) scores under laboratory and ambulatory conditions were evaluated by Pearson correlation, which were interpreted as follows: values between 0.1 and 0.69 denoted weak to moderate correlation, values above 0.7 were regarded as a strong correlation [27]. Friedman’s test, with post hoc Wilcoxon Signed-rank tests, were used to evaluate the change in pain and fatigue NRS scores across test sessions. SPSS for Windows version 26 (IBM, Armonk, New York, USA), MedCalc version 19.5.1 (MedCalc Software, Ostend, Belgium) and Microsoft Excel for Windows version 2013 (Microsoft Corporation, Redmond, Washington, USA) were used for analysis.

## 3. Results

### 3.1. Recruitment and Participant Characteristics

**Figure 2:**
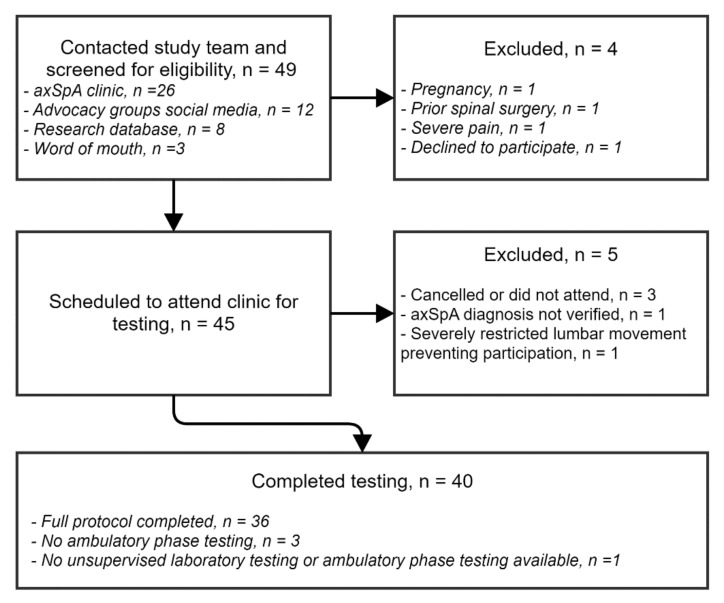
Participant recruitment.

Forty eligible participants were recruited to the study and completed the protocol between April 2018 and December 2018. Figure 2 illustrates the participant recruitment to the study. Twenty-five participants were male and 15 were female. Mean age of participants was 48.0 years (range 27 to 76) and mean symptom duration was 23.6 years (range 3 to 52). A range of disease severity is seen in the scores across clinical measures. Sixty-five percent of the participants were taking anti-TNFα medication. Participant baseline characteristics are summarized in Table 2.

### 3.2. Protocol Fidelity

Thirty-six participants completed the study as per full protocol. One participant completed supervised testing but did not participate in the unsupervised laboratory testing or the ambulatory phase of testing due to a flare-up of leg pain. Technical issues affected three ambulatory test sessions; one sensor malfunctioned, one sensor fell off and was incorrectly re-positioned by the participant, and one recorder had insufficient battery for data collection. In all of these cases, the data was lost. During the unsupervised conditions, five participants performed an incorrect number of movement repetitions and one participant did not perform the movements bilaterally. Two participants did not consistently use the event button to record the start and end of a movement; this made identification of the tests difficult for the data analyst, as their movement was restricted and no clear movement sequence could be identified from the raw data.

### 3.3. Spinal Mobility Data

The ‘full-arc’ ROM of each measurement using the IMU sensors are summarized in Table 3. The normalized indices for each measurement, the BASMI_Lin_ and the composite IMU-ASMI (Amb) scores are summarized in Table 4.

### 3.4. Reliability and Agreement of IMU Movements

The test-retest reliability results for IMU movement tests performed in the laboratory are summarized in Table 5. Both the Trunk IMU and Lumbar region IMU showed good to excellent agreement for all movements. The SEM ranged from 5.12° to 9.02° for Flexion + Extension, 2.12° to 4.86° for Lateral flexion, and 5.98° to 8.19° for Rotation (see Supplementary Table S1). Test-retest reliability and agreement of IMU movement tests performed on different days are available in Supplementary Table S2. 

The reliability and agreement analyses of IMU movement tests performed under laboratory and ambulatory conditions are summarized in Table 6. Both the Trunk IMU and Lumbar region IMU showed good to excellent agreement for all movements. The SEM ranged from 4.67° to 8.54° for Flexion + Extension and 2.17° to 5.39° for lateral flexion.

### 3.5. Reliability and Agreement of IMU-ASMI (Amb) Indices

The reliability and agreement analyses of IMU-ASMI (Amb) scores are summarized in Table 5 and Table 6. Both the Trunk-ASMI (Amb) and Lumbar-ASMI (Amb) showed strong agreement under laboratory and ambulatory conditions. The SEM ranged from 0.36 to 0.77 for Trunk-ASMI (Amb) and 0.43 to 0.69 for Lumbar-ASMI (Amb). The IMU-ASMI (Amb) scores showed good correlation with BASMI_Lin_ under all test conditions (see Table 7). Pearson correlations were ≥0.85.

### 3.6. Pain and Fatigue Monitoring 

Thirty-three participants completed self-report pain and fatigue NRS during all three test sessions (Day 1, Ambulatory, Day 2). There was a statistically significant difference in fatigue NRS scores depending on the test session, χ^2^(2) = 8.6154, *p* < 0.001. Post hoc analysis with Wilcoxon signed-rank test was conducted with a Bonferroni-adjusted significance level set at *p <* 0.017. Median (IQR) fatigue NRS scores for Day 1, Ambulatory and Day 2 sessions were 3.0 (1.8 to 6.0), 4.0 (2.0 to 6.3), and 3.0 (1.0 to 5.0), respectively. There was a statistically significant reduction in fatigue score on Day 2 compared to the ambulatory session (*Z* = 3.0567, *p* = 0.0022). No statistically significant differences in fatigue NRS scores were observed between Day 1 and ambulatory (*Z* = −1.20, *p* = 0.23) or Day 2 sessions (between *Z* = 1.20, *p* = 0.1192). No statistically significant effect of test session on pain NRS scores was observed, χ^2^ (2) = 0.1538, *p* = 0.86. Median (IQR) pain NRS scores for Day 1, Ambulatory and Day 2 sessions were 2.0 (1.0 to 3.3), 2.0 (0.8 to 3.0), and 2.0 (0.0 to 3.0), respectively.

## 4. Discussion

This study demonstrates the reliability of an IMU sensor-based system for measuring spinal range of motion of individuals with axSpA. The IMU sensor-based system showed good to excellent test-retest reliability under supervised and unsupervised conditions in the laboratory setting, and unsupervised in the home setting.

Composite IMU-ASMI (Amb) scores were calculated for the lumbopelvic region (Trunk-ASMI) and the lumbar region (Lumbar-ASMI) based on methods used in previous studies [19,24]. In this study, rotation movement data was only included in the supervised IMU-ASMI (Amb) scores due to technical limitations within the system. As rotation has a smaller range of movement in the lumbar spine than movement in the other two planes, this limitation was hypothesized to have been of negligible practical consequence [28,29,30]. Reliability of composite IMU-ASMI (Amb) scores was excellent across supervised test conditions, with ICCs for IMU-ASMI (Amb) scores under supervised conditions similar to previously reported scores in a similar cohort [19]. Reliability of composite IMU-ASMI (Amb) scores was also found to be excellent in unsupervised ambulatory settings; an unsupervised IMU-ASMI (Amb) score could therefore function as a reliable surrogate for a supervised IMU-ASMI score.

The limits of agreement showed greater full-arc range of motion and lower IMU-ASMI (Amb) scores (better performance) when participants were supervised than when unsupervised, suggestive of a small systematic bias. Participants may have tried harder when under direct observation than when unsupervised, due to beliefs about researcher expectations and social desirability [31]. Participants may also be more likely to perform movements slightly ‘off-plane’ or with less accuracy when performing the movements unsupervised, resulting in reduced range of motion being recorded. Circadian rhythm of symptoms in axSpA may have influenced the performance of spinal movement tasks, however, pain and fatigue symptoms were shown to be largely stable across test sessions.

A secondary aim of the study was to evaluate the reliability of an IMU-ASMI (Amb) score and to determine correlation with conventional metrology. Both supervised and unsupervised IMU-ASMI (Amb) scores showed strong correlations with BASMI_Lin_ and may be a suitable proxy for conventional metrology when direct measurement by a clinician is not possible. A limitation of the IMU-ASMI (Amb) scores described is they do not include measures of standing posture, hip or neck range of motion. As a result, they should not be considered a substitute for conventional BASMI. Including these additional components would require additional IMU sensors, and longer set-up and test protocol time and was beyond the scope of this study. Despite this limitation, the IMU-ASMI (Amb) gives a comprehensive and accurate representation of spinal movement in degrees across three planes of movement.

This study supports the concept that individuals with axSpA can use an IMU sensor-based system to monitor their spinal mobility reliably and accurately, without supervision at home or in non-clinical settings. While this would not replace supervised tests in a clinical setting, it offers clinicians a reliable method of remotely monitoring spinal mobility in individuals with axSpA. This is an important step in developing a system that will allow clinicians and researchers to track small changes in spinal mobility over time, and measure the impact of exercise programs, without necessitating frequent, in-person consultations. The increase in remote telehealth consultations, accelerated by the SARS-CoV-2 pandemic, is broadly supported by patients and clinicians [12,32,33,34]. However, the inability to conduct a physical examination of spinal mobility has presented a persistent obstacle to the adoption of remote consultations [33,35]. IMU sensor-based systems could provide a solution by facilitating reliable and accurate measurement of spinal metrology.

eHealth and mobile-based applications have been recognized as potential ways of improving remote monitoring. Mobile health (mHealth) can contribute to the empowerment of patients, who could manage their health more actively and live more independently thanks to self-assessment or remote monitoring solutions, and support healthcare professionals in treating patients more efficiently [36]. Most studies to date examining eHealth and mHealth in rheumatology have focused on rheumatoid arthritis [37,38]. Recent systematic reviews have identified approximately 35 apps currently available that offer symptom tracking, educational information and links to online communities for people with rheumatic and musculoskeletal disease [39,40,41]. However, only one app was specific to axSpA [42]. Future development of an IMU sensor-based system linked to a mobile application could enhance the utility and specificity of such an application in relation to axSpA, where monitoring of spinal range of motion is an important indicator of disease progression [43,44].

In addition to providing data to a clinician, the output of the IMU sensor-based system could support self-management interventions [45,46,47,48]. IMU-ASMI (Amb) scores, expressed on a scale of 0 to 10, are easy to interpret without knowledge of the normal ranges of each spinal movement. This could be used by people with axSpA as a motivational point to encourage adherence to exercise or pharmaceutical treatments and facilitate self-monitoring during maintenance phases or disease flares. It is a strength of the study that a broad cross-section of individuals with axSpA participated, ranging in demographic characteristics, clinical features and treatments. The majority of participants completed the simple test movements unsupervised by following simple standardized instructions, either by video or written instructions; just ten percent of participants failed to complete the study protocol in full, demonstrating the feasibility.

One participant did not complete the ambulatory phase testing as a sensor detached from the skin, and they were unable to reposition it themselves. This illustrates a limitation of the current sensor setup, with participants unable to self-attach sensors with the degree of precision required. In this study sensors were attached by a trained member of the research team, however, if this is to be adopted as a self- or remote-monitoring tool, an alternate way of attaching sensors is required. This study used a two-sensor setup, however, a single sensor set-up warrants further investigation; results from the Trunk IMU (positioned at L1 vertebra) showed equivalent reliability to the results from the two sensor Lumbar region IMU setup. Finally, unlike conventional metrology measures, the sensor set-up used in this study does not include measures of cervical mobility. However, the correlation between mean IMU–ASMI (Amb) versus mean BASMI_Lin_ was 0.82, which suggests that the IMU–ASMI (Amb) is a clinically relevant measure, despite not including the cervical region. Furthermore, it has been previously shown that removing cervical mobility tests does not affect the reliability of the IMU-ASMI [19].

Currently, standard clinical tests of spinal movement in individuals with axSpA focus on movements in a single plane. IMU sensor systems offer the potential for measuring multi-planar spinal movements that would be closer to ‘real-world’ movements; as well as providing the additional benefit of monitoring and attaining data over longer periods than that of a clinic-based assessment. Future research should seek to establish the validity and reliability of an IMU sensor-based system to measure spinal mobility during functional movement tests. Performance-based tests may be of more interest to both the clinician and the patient and may be a more objective measure of function instead of pure mobility.

## 5. Conclusions

This study has demonstrated that an IMU sensor-based system is a reliable way of measuring spinal mobility in axSpA under supervised and unsupervised conditions. While not a replacement for established clinical measures, composite IMU-ASMI (Amb) scores may be reliably used as a proxy measure of spinal mobility.

## Figures and Tables

**Table 1:** Study assessment schedule.

	Day 1—Laboratory	Ambulatory Phase	Day 2—Laboratory
Baseline data collection	√	—	—
BASMI_Lin_ and chest expansion	√	—	√
Pain NRS and Fatigue NRS	√	√	√
Spinal movement tests	Supervised and Unsupervised	Unsupervised	Supervised and Unsupervised

BASMI_Lin_: Bath Ankylosing Spondylitis Metrology Index (linear version); NRS: numeric rating scale.

**Table 2:** Descriptive characteristics of study participants.

Variable	AxSpA Cohort (*n* = 40)
Age, years	48.0 (12.9); [27–76]
Sex (male/female), *n*	25/15
Symptom duration, years	23.6 (13.7); [3–52]
Time since diagnosis, years	9.0 (26.5) *; [0–43]
BMI, kg/m^2^	28.4 (7.5) *; [20.0–37.7]
Employed, *n* (%)	23 (57.5)
BASMI_Lin_^†^	3.8 (1.8); [1.2–7.9]
Lateral lumbar flexion	4.9 (2.5); [0–9.0], (2.3cm–21.5cm) ^‡^
Tragus to wall distance	2.4 (2.0); [0.5–7.6], (9.5cm–30.8cm) ^‡^
Modified Schober’s test	5.4 (2.5); [0–9.7], (0.6cm–13.3cm) ^‡^
Intermalleolar distance	3.1 (1.9); [0–7.0], (55.0cm–138.5cm) ^‡^
Cervical rotation	3.3 (2.2); [0.3–9.5], (9.0°–87.0°) ^‡^
Chest expansion, cm	2.5 (2.2); [0.6–13.2]
Pharmacology, *n* (%)	
Anti-TNFα	26 (65)
NSAIDs	4 (10)
Analgesia	4 (10)
None	6 (15)
HLA-B27 status, *n* (%)	
Positive	17 (42.5)
Negative	7 (17.5)
Unknown	16 (40)
BAS-G, (scale 0–10)	3.4 (2.1) [0–7]
BASDAI ^§^, (scale 0–10)	3.4 (2.0) [0–7.9]
BASFI, (scale 0–10)	3.4 (2.4) [0–8.4]

Results are presented as mean (SD); [min-max] unless otherwise stated. * Median (IRQ). ^†^ BASMI_Lin_ values from initial assessment. BASMI_Lin_ component results are item values on a 0—10 scale. The higher the BASMI_Lin_ score, the more severe the individual’s limitation of movement. ^‡^ Min-max scores in original units of measurement are shown in brackets. ^§^ BASDAI not completed by *n* = 1 participant. Abbreviations—BAS-G: Bath Ankylosing Spondylitis Global Score; BASDAI: Bath Ankylosing Spondylitis Disease Activity Index; BASFI: Bath Ankylosing Spondylitis Functional Index; BASMI_Lin_: Bath Ankylosing Spondylitis Metrology Index (linear version); BMI: body mass index.

**Table 3:** Range of movement of participants measured by IMU sensors.

Method	Movement	Day 1—Supervised *	Day 1—Unsupervised ^†^	Ambulatory ^‡^	Day 2—Supervised *	Day 2—Unsupervised ^†^
Trunk IMU	Flexion-Extension	125.7 (27.1)	121.0 (27.2)	120.1 (27.4)	123.7 (25.6)	121.4 (26.4)
	Lateral flexion L + R	46.8 (19.7)	45.4 (17.8)	43.9 (18.2)	47.1 (19.8)	45.5 (19.0)
	Rotation L + R	42.1 (22.2)	-	-	42.2 (22.4)	-
Lumbar region IMU	Flexion-Extension	60.9 (27.0)	58.6 (26.2)	57.8 (25.4)	58.2 (26.4)	56.6 (24.9)
	Lateral flexion L + R	35.4 (19.1)	34.3 (19.2)	33.6 (19.1)	35.4 (19.2)	34.4 (19.0)
	Rotation L + R	27.1 (16.1)	-	-	26.8 (15.9)	-

Figures presented as mean (SD). All movements in degrees (°). * *n* = 40; ^†^ *n* = 39; ^‡^ *n* = 36. Trunk IMU: the orientation angle from the upper lumbar sensor to the ground; represents lumbar and pelvic movement. Lumbar region IMU: the angle between the upper sensor and the sacral sensor; represents lumbar movement. Output for rotation movements was only available under supervised conditions owing to technical limitations with the system.

**Table 4:** Normalized indices for each IMU movement and composite IMU-ASMI (Amb) score per IMU region.

Method	Movement	Day 1—Supervised *	Day 1—Unsupervised ^†^	Ambulatory ^‡^	Day 2—Supervised *	Day 2—Unsupervised ^†^
Trunk IMU	Flexion-Extension	2.2 (2.0)	2.6 (2.0)	2.7 (2.0)	2.3 (2.0)	2.5 (2.1)
	Lateral flexion L + R	4.0 (2.7)	4.2 (2.4)	4.4 (2.5)	4.0 (2.7)	4.2 (2.6)
	Rotation L + R	3.4 (3.2)	-	-	3.4 (3.2)	-
	Trunk-ASMI (Amb)	3.2 (2.3)	3.4 (2.1)	3.5 (2.1)	3.2 (2.3)	3.3 (2.2)
Lumbar region IMU	Flexion-Extension	2.5 (2.9)	2.6 (3.0)	2.7 (2.9)	2.7 (3.1)	2.8 (3.0)
	Lateral flexion L + R	4.1 (3.3)	4.3 (3.3)	4.4 (3.3)	4.1 (3.3)	4.3 (3.2)
	Rotation L + R	3.1 (3.3)	-	-	3.0 (3.2)	-
	Lumbar-ASMI (Amb)	3.2 (2.8)	3.5 (3.0)	3.5 (3.0)	3.2 (2.9)	3.5 (3.0)
BASMI_Lin_	Total score	3.8 (1.8)	-	-	3.8 (1.8)	-

Figures presented as mean (SD). * *n* = 40; ^†^ *n* = 39; ^‡^ *n* = 36. Trunk IMU: the orientation angle from the upper lumbar sensor to the ground; represents lumbar and pelvic movement. Lumbar region IMU: the angle between the upper sensor and the sacral sensor; represents lumbar movement. Output for rotation movements was only available under supervised conditions owing to technical limitations with the system. Abbreviations—BASMI_Lin_: Bath Ankylosing Spondylitis Metrology Index (linear version).

**Table 5:** Test-retest reliability and agreement of full-arc movement measurements and composite – ASMI (Amb) scores under supervised and unsupervised conditions in the laboratory.

	Supervised Day 1 v Supervised Day 2 *					Unsupervised Day 1 v Unsupervised Day 2 ^†^					Supervised Day 1 v Unsupervised Day 1 ^†^					Supervised Day 2 v Unsupervised Day 2 ^†^				
	ICC [95% CI]	SEM	95% LOA			ICC [95% CI]	SEM	95% LOA			ICC [95% CI]	SEM	95% LOA			ICC [95% CI]	SEM	95% LOA		
			Bias	Lwr	Upr			Bias	Lwr	Upr			Bias	Lwr	Upr			Bias	Lwr	Upr
**Trunk** **IMU**																				
Flexion + Extension	**0.93** [0.88–0.96	6.98	2.0	−16.7	20.6	**0.93** [0.88–0.96]	7.20	−0.3	−19.5	18.8	**0.96** [0.84–0.98]	5.45	4.7	−7.6	17.1	**0.94** [0.90–0.97]	6.00	2.4	−14.1	19.0
Lateral flexion L + R	**0.96** [0.93–0.98]	3.95	−0.3	−11.4	10.8	**0.95** [0.91–0.97]	3.99	−0.0	−11.5	11.4	**0.94** [0.89–0.97]	4.86	2.5	−18.6	23.7	**0.97** [0.94–0.98]	3.45	2.8	−13.4	19.0
Trunk-ASMI (Amb)	**0.94** [0.89–0.97]	0.56	−0.0	−1.6	1.5	**0.96** [0.94–0.98]	0.42	0.0	−1.15	1.22	**0.91** [0.84–0.95]	0.68	−0.2	−2.0	1.5	**0.93** [0.86–0.96]	0.62	−0.1	−1.9	1.6
**Lumbar region IMU**																				
Flexion + Extension	0.89 [0.80–0.94]	9.02	2.7	−21.8	27.2	0.89 [0.80–0.94]	8.70	2.0	−21.4	25.4	**0.96** [0.93–0.98]	5.12	3.8	−28.4	35.9	**0.98** [0.97–0.99]	3.57	0.9	−8.7	10.5
Lateral flexion L + R	**0.98** [0.96–0.99]	2.84	−0.1	−8.1	7.9	**0.97** [0.95–0.99]	3.32	−0.1	−8.6	8.5	**0.98** [0.97–0.99]	2.45	0.7	−6.1	7.5	**0.99** [0.98–0.99]	2.12	0.9	−4.7	6.6
Lumbar-ASMI (Amb)	**0.95** [0.90–0.97]	0.63	−0.0	−1.9	1.8	**0.96** [0.93–0.98]	0.60	−0.1	−1.64	1.50	**0.96** [0.92–0.98]	0.57	−0.3	−1.9	1.4	**0.96** [0.92–0.98]	0.57	−0.3	−1.9	1.4

All ICC results were statistically significant, *p* < 0.001. Bold denotes ICC > 0.9. * *n* = 40; ^†^ *n* = 39. Abbreviations—ICC: Intraclass correlation coefficient; SEM: standard error of measurement (deg); 95% LOA: 95% limits of agreements (deg).

**Table 6:** Test-retest reliability and agreement of full-arc movement measurements and composite –ASMI (Amb) scores under laboratory and ambulatory conditions.

	Supervised Day 1 v Ambulatory					Unsupervised Day 1 v Ambulatory					Supervised Day 2 v Ambulatory					Unsupervised Day 2 v Ambulatory				
	ICC [95% CI]	SEM	95% LOA			ICC [95% CI]	SEM	95% LOA			ICC [95% CI]	SEM	95% LOA			ICC [95% CI]	SEM	95% LOA		
			Bias	Lwr	Upr			Bias	Lwr	Upr			Bias	Lwr	Upr			Bias	Lwr	Upr
**Trunk IMU**																				
Flexion + Extension	**0.94** [0.58–0.98]	6.56	6.9	−6.1	19.9	**0.97** [0.94–0.98]	4.67	2.3	−10.0	14.6	0.89 [0.78–0.94]	8.54	4.7	−18.6	28.0	**0.92** [0.85–96]	7.52	2.4	−18.5	23.3
Lateral flexion L + R	**0.94** [0.84–0.97]	4.95	7.3	−19.4	34.1	**0.96** [0.93–0.98]	3.31	4.8	−18.1	27.7	**0.93** [0.81–0.97]	5.39	7.6	−18.8	34.0	**0.97** [0.93–0.98]	3.45	4.8	−18.8	28.4
Trunk-ASMI (Amb)	**0.91** [0.80–0.96]	0.68	−0.4	−2.1	1.3	**0.97** [0.94–0.99]	0.36	−0.2	−1.1	0.6	0.89 [0.78–0.94]	0.77	−0.3	−2.4	1.7	**0.97** [0.93–0.98]	0.39	−0.2	−1.2	0.7
**Lumbar region IMU**																				
Flexion + Extension	**0.93** [0.86–0.96]	7.18	3.6	−14.7	21.8	**0.92** [0.85–0.96]	7.52	2.1	−18.2	22.5	**0.93** [0.87–0.96]	6.82	0.5	−18.3	19.2	**0.93** [0.88–0.97]	6.38	−0.1	−18.2	17.9
Lateral flexion L + R	**0.98** [0.94–0.99]	2.73	2.0	−4.6	8.7	**0.98** [0.97–0.99]	2.33	1.2	−4.9	7.4	**0.97** [0.93–0.98]	3.43	2.1	−6.8	10.9	**0.98** [0.96–0.99]	2.61	1.1	−6.1	8.2
Lumbar-ASMI (Amb)	**0.94** [0.88–0.97]	0.69	−0.4	−2.2	1.4	**0.97** [0.93–0.98]	0.52	−0.2	−1.7	1.3	**0.95** [0.90–0.97]	0.64	−0.3	−2.0	1.5	**0.98** [0.97–0.99]	0.43	−0.1	−1.1	1.0

All ICC results were statistically significant, *p* < 0.001. *n* = 36. Bold denotes ICC > 0.9. Abbreviations—ICC: Intraclass correlation coefficient; SEM: standard error of measurement (deg); 95% LOA: 95% limits of agreements (deg).

**Table 7:** Pearson correlations between BASMI_Lin_ and IMU-ASMI (Amb) scores under laboratory and ambulatory conditions.

Method	Test	BASMI_Lin_ Day 1	BASMI_Lin_ Day 2
Trunk-ASMI (Amb)	Supervised Day 1	0.85	0.87
	Supervised Day 2	0.85	0.88
	Unsupervised Day 1	0.85	0.88
	Unsupervised Day 2	0.86	0.91
	Ambulatory	0.88	0.91
Lumbar-ASMI (Amb)	Supervised Day 1	0.86	0.88
	Supervised Day 2	0.86	0.86
	Unsupervised Day 1	0.86	0.88
	Unsupervised Day 2	0.86	0.90
	Ambulatory	0.87	0.89

Abbreviations—BASMI_Lin_: Bath Ankylosing Spondylitis Metrology Index (linear version).

## Data Availability

The data presented in this study are available on request from the corresponding author. The data are not publicly available due to ethical restrictions.

## Supplementary Materials

The following are available online at https://www.mdpi.com/2075-4418/11/3/490/s1, Supplementary Table S1: Test-retest reliability and agreement of full-arc rotation measurements between IMU sensors under supervised conditions in the laboratory; Supplementary Table S2: Test-retest reliability and agreement of full-arc movement measurements and composite -ASMI (Amb) scores under supervised and unsupervised conditions on different days in the laboratory, Supplementary Document: The BASMI_Lin_ and IMU-ASMI composite indices explained in detail; Supplementary Video: Example of instructional video.
