# Supplementary material for: Measuring Spinal Mobility Using an Inertial Measurement Unit System: A Reliability Study in Axial Spondyloarthritis
Source: Diagnostics (Basel). 2021 Mar 10;11(3):490. doi: 10.3390/diagnostics11030490 (PMC8001996; doi:10.3390/diagnostics11030490)
Supplement: Supplementary file 1 [file diagnostics-11-00490-s001.zip › diagnostics-1127178/Supplemental Table S1.pdf]

Supplemental Table S1. Test-retest reliability and agreement of full-arc rotation measurements between IMU sensors under supervised conditions in the laboratory

	**Supervised Day 1 v Supervised Day 2**				
	ICC [95% CI]	SEM	Bias	95% LOA Lwr	95% LOA Upr
	**Rotation L+R**				
Trunk IMU	0.86 [0.76-0.93]	8.19	−0.2	−23.2	22.9
Lumbar region IMU	0.86 [0.75-0.92]	5.98	0.3	−16.3	16.9

n = 40. All ICC results were statistically significant, $p < 0.001$. Abbreviations—ICC: Intraclass correlation coefficient; SEM: standard error of measurement (deg); 95% LOA: 95% limits of agreements (deg)

Supplemental Table S2. Test-retest reliability and agreement of full-arc movement measurements and composite -ASMI (Amb) scores under supervised and unsupervised conditions on different days in the laboratory

	**Supervised Day 1 v Unsupervised Day 2**					**Unsupervised Day 1 v Supervised Day 2**				
	ICC [95% CI]	SEM	Bias	95% LOA Lwr	95% LOA Upr	ICC [95% CI]	SEM	Bias	95% LOA Lwr	95% LOA Upr
				**Trunk IMU**						
Flexion + Extension	**0.93** [0.84-0.96]	7.31	4.4	−14.1	22.9	**0.91** [0.84-0.95]	8.17	−2.8	−24.1	18.6
Lateral flexion L+R	**0.95** [0.91-0.98]	4.28	2.5	−17.9	23.0	**0.92** [0.85-0.96]	5.04	−2.8	−22.7	17.0
Trunk-ASMI (Amb)	**0.92** [0.86-0.96]	0.64	−0.2	−1.9	1.6	0.88 [0.78-0.93]	0.72	0.2	−2.0	2.3
				**Lumbar region IMU**						
Flexion + Extension	**0.90** [0.82-0.95]	8.37	3.4	−18.1	24.9	0.87 [0.76-0.93]	9.46	1.1	−25.7	27.8
Lateral flexion L+R	**0.98** [0.96-0.99]	2.92	0.6	−7.5	8.7	**0.96** [0.93-0.98]	3.84	−1.0	−11.3	9.3
Lumbar-ASMI (Amb)	**0.94** [0.89-0.97]	0.69	−0.3	−2.2	1.5	**0.92** [0.85-0.96]	0.85	0.2	−2.1	2.5

n = 39. All ICC results were statistically significant, $p < 0.001$. Bold denotes ICC >0.9. Abbreviations—ICC: Intraclass correlation coefficient; SEM: standard error of measurement (deg); 95% LOA: 95% limits of agreements (deg)

Supplementary Document: The $BASMI_{Lin}$ and IMU-ASMI composite indices explained in detail

1.0 $BASMI_{Lin}$

The linear Bath Anklyosing Spinal Metrology Index ($BASMI_{Lin}$) is a composite index intended to represent mobility in axial spondyloarthritis (axSpA) [26]. It includes two measures of movement/deformity in the cervical spine (tragus-to-wall distance, cervical rotation), two tests of mobility in the lumbar spine (modified Schober's test, lateral flexion) and one of hip mobility (intermalleolar distance). Four of the five measurements use units of distance (cm), while one is measured in degrees (cervical rotation). Limitations of the tool are that distance based measurements are more prone to variation in normal individuals, purely based on height. Additionally, rotation in the lumbar/thoracic spine is not assessed, and these tests cannot be performed independently.

The absolute measurements in the $BASMI_{Lin}$ vary from quite small ranges e.g. Schober's test 0.7cm–>7.0cm, to large e.g. cervical rotation ≤8.5–≥85 degrees. These values are 'normalised' to a 0–10 dimensionless scale, where 0 represents normal range of motion (ROM) and 10 represents the most severely restricted ROM. The scale ranges used in the formulae for the $BASMI_{Lin}$ (see Table 1) are based on the maximum range of motion measured in population samples that include both normal individuals (highest decile) and those with severe axSpA (lowest decile). The scale between these points is then divided into 10 equal units. When each component is normalised, they can be compared with each other on an equivalent scale. They can also be combined into a composite score by taking the average (the $BASMI_{Lin}$).

Table 1. Equations used for the conversion of the assessments (*A*) into scores (*S*) for the five components of the $BASMI_{Lin}$ (taken from van der Heijde et al. 2008) (26)

	**BASMI component**	***S* = 0 if:**	**Between 0 and 10:**	***S* = 10 if:**
**A**	Lateral lumbar flexion* (cm)	$A \geq 21.1$	$S = (21.12 - A)/2.1$	$A \leq 0.1$
**B**	Tragus-to-wall distance* (cm)	$A \leq 8$	$S = (A - 8)/3$	$A \geq 38$
**C**	Lumbar flexion (modified Schober's test) (cm)	$A \geq 7.4$	$S = (7.42 - A)/0.7$	$A \leq 0.4$
**D**	Intermalleolar distance (cm)	$A \geq 124.5$	$S = (124.5 - A)/10$	$A \leq 24.5$
**E**	Cervical rotation angle* (degrees)	$A \geq 89.3$	$S = (89.3 - A)/8.5$	$A \leq 4.3$

* For lateral lumbar flexion, tragus-to-wall distance, and cervical rotation the average of right and left should be taken. Note that 'half-arc' ranges are used for cervical rotation and lateral lumbar flexion.

$BASMI_{Lin}$ Formula: (A+B+C+D+E)/5

2.0 The IMU-ASMI

The full IMU-ASMI was intended to be a comprehensive spinal mobility test using wearable inertial motion unit (IMU) sensors, carried out under supervised conditions in the laboratory. It includes three planar range of motion tests for the cervical spine and three for the lumbar spine (see Table 2). The average of all six measurements is used to calculate the full IMU-ASMI, but it is also possible to split it into lumbar and cervical components without loss of reliability (19).

The scale ranges used in the formulae for the IMU-ASMI (see Table 2) are based on the maximum range of motion measured in population samples that include both normal individuals (highest decile) and those with severe axSpA (lowest decile), and is based on that of the $BASMI_{Lin}$. The population sample (collected by the project group and used throughout the studies within this

project) for the reference range was small, so the upper limits may be refined in future as more normal subjects are studied.

Table 2. Equations used for the conversion of the assessments (*A*) into scores (*S*) for the components of the Trunk IMU-ASMI and Lumbar IMU-ASMI.

	Spinal Segment	Movement (recorded by sensor)*	*S* = 0 if:	Between 0 and 10:	*S* = 10 if:
**A**	Lumbar	Flexion/Extension (Trunk)	*A*>149*	*S* = (149−A)/11.6	*A*≤33*
**B**		Flexion/Extension (Lumbar region)	*A*>72*	*S* = (72−A)/6.9	*A*≤3*
**C**		Lateral Flexion (Trunk)	*A*>76*	*S* = (76−A)/7.3	*A*≤3*
**D**		Lateral Flexion (Lumbar region)	*A*>58*	*S* = (58−A)/5.6	*A*≤2*
**E**		Rotation (Trunk)	*A*>59*	*S* = (59−A)/5.8	*A*≤1*
**F**		Rotation (Lumbar region)	*A*>33*	*S* = (33−A)/3.3	*A*≤0*
**G**	Cervical	Flexion/Extension	*A*>107*	*S* = (107−A)/10.3	*A*≤4*
**H**		Lateral Flexion	*A*>77*	*S* = (77−A)/7.7	*A*≤0*
**I**		Rotation	*A*>150*	*S* = (150−A)/14.2	*A*≤8*

*Measured in degrees. Note that 'full-arc' ranges are used for IMU-ASMI calculations. Trunk: the orientation angle from the upper lumbar sensor to the ground; represents lumbar and pelvic movement. Lumbar region: the angle between the upper sensor and the sacral sensor; represents lumbar movement.

Trunk IMU-ASMI Formula: (A+C+E+G+H+I)/6—using data from single sensor (Trunk)
Lumbar IMU-ASMI Formula: (B+D+F+G+H+I)/6—using data from two sensors (Trunk and Lumbar region)

3.0 The Ambulatory IMU-ASMI

The ambulatory version, IMU-ASMI (Amb), is designed to be used in an unsupervised, ambulatory setting e.g. at home or at work. As such, some of the movements included in the original IMU-ASMI were not included. For example, when consulting with individuals with axSpA, we were advised that if the sensors were to be worn all day they would need to be unobtrusive – and that most people would not be willing to wear a head mounted sensor to measure neck movement. Therefore, only the lumbar spinal segment could be assessed. The IMU-ASMI (Amb), performed under supervised conditions in the laboratory (see *Section 2.4: Assessment Schedule*), is the average of the three normalised movements as described in Table 2. This version compares closely with the IMU-ASMI used by Gardiner et al. (19).

Supervised Trunk IMU-ASMI (Amb) Formula: (A+C+E)/3—using data from single sensor (Trunk)
Supervised Lumbar IMU-ASMI (Amb) Formula: (B+D+F)/3—using data from two sensors (Trunk and Lumbar region)

When lumbar rotation tests are performed in the supervised setting, the tester has to press the spacebar on the laptop after each rotation movement to 'reset' the baseline. This is due to 'drift' in the gyroscope reading, which continues after the movement has stopped. This intervention is not needed for other movement tests, so they were considered more practical and useful for ambulatory tests. Additionally, rotation is minimal in the lumbar spine (see *Section 4: Discussion*). Therefore, we opted to focus on two important lumbar movements, flexion/extension and lateral flexion. The IMU-ASMI

(Amb) performed under unsupervised conditions in the laboratory and ambulatory settings, is the average of the two normalised movements.

Unsupervised Trunk IMU-ASMI (Amb) Formula: (A+C)/2—using data from single sensor (Trunk)
Unsupervised Lumbar IMU-ASMI (Amb) Formula: (B+D)/2—using data from two sensors (Trunk and Lumbar region)

The regions measured by each index ($BASMI_{Lin}$, IMU-ASMI, supervised IMU-ASMI (Amb) and unsupervised IMU-ASMI (Amb)), as well as the range of motion and functional importance associated with each, are summarized in Table 3.

Table S3: Regions represented in the movement indices

**Region**	**Movement**	**ROM***	**Functional importance†**	**BASMI$_{Lin}$**	**IMU-ASMI‡**	**Super ASM**
Cervical spine	Flexion/Extension	++	++	Tragus-to-wall distance	++	
	Lateral Flexion	+	+	N/A	+	
	Rotation	+++	+++	Cervical rotation angle	+++	
Thoracic spine	Rotation	+++	+++	N/A	N/A§	
Lumbar spine	Flexion/Extension	+++	+++	Modified Schober's test	+++	
	Lateral Flexion	++	++	Lateral lumbar flexion	++	
	Rotation	+	+	N/A	+	
Pelvis	Flexion/Extension	+	+	N/A	+	
	Lateral Flexion	+	+	N/A	+	
	Rotation	+	+	N/A	+	
Hip	Abduction	++	+++	Intermalleolar distance	N/A	

*+—Small amount of movement ++—moderate amount of movement +++—large amount of movement. †+—Small amount of importance ++—moderate amount of importance +++—large amount of importance. ‡+, ++ and +++ indicates the relative size of each range of movement test incorporated in the index. Pelvis movement is taken into account in the two-sensor lumbar IMU-ASMI. §Future tests may permit accurate rotation measurements in the thoracic spine for inclusion in IMU-ASMI. Abbreviations—ROM: Range of motion; BASMI$_{Lin}$: Bath Ankylosing Spondylitis Metrology Index (linear version); IMU-ASMI: Inertial Motion Unit Ankylosing Spondylitis Metrology Index; IMU-ASMI (Amb): Inertial Motion Unit Ankylosing Spondylitis Metrology Index Ambulatory Version; N/A: Not assessed.
