# Supplementary material for: Measuring Spinal Mobility Using an Inertial Measurement Unit System: A Reliability Study in Axial Spondyloarthritis
Source: Diagnostics (Basel). 2021 Mar 10;11(3):490. doi: 10.3390/diagnostics11030490 (PMC8001996; doi:10.3390/diagnostics11030490)
Supplement: Supplementary file 1 [file diagnostics-11-00490-s001.zip › Supplemental Table S1.docx]

**Supplemental Table S1.** Test-retest reliability and agreement of full-arc rotation measurements between IMU sensors under supervised conditions in the laboratory

|  | Supervised Day 1 v Supervised Day 2 | | | | | |  |
| --- | --- | --- | --- | --- | --- | --- | --- |
|  | ICC [95% CI] | SEM | 95% LOA | | |  |  |
|  |  |  | Bias | Lwr | Upr |  |  |
| **Rotation L+R** | | | | | | | |
| Trunk IMU | 0.86 [0.76-0.93] | 8.19 | −0.2 | −23.2 | 22.9 |  |  |
| Lumbar region IMU | 0.86 [0.75-0.92] | 5.98 | 0.3 | −16.3 | 16.9 |  |  |

*n* = 40. All ICC results were statistically significant, *p* < 0.001. Abbreviations—ICC: Intraclass correlation coefficient; SEM: standard error of measurement (deg); 95% LOA: 95% limits of agreements (deg)
