# Supplementary material for: Measuring Spinal Mobility Using an Inertial Measurement Unit System: A Reliability Study in Axial Spondyloarthritis
Source: Diagnostics (Basel). 2021 Mar 10;11(3):490. doi: 10.3390/diagnostics11030490 (PMC8001996; doi:10.3390/diagnostics11030490)
Supplement: Supplementary file 1 [file diagnostics-11-00490-s001.zip › Supplemental Table S2.docx]

**Supplemental Table S2.** Test-retest reliability and agreement of full-arc movement measurements and composite -ASMI (Amb) scores under supervised and unsupervised conditions on different days in the laboratory

|  | Supervised Day 1 v Unsupervised Day 2 | | | | | Unsupervised Day 1 v Supervised Day 2 | | | | |
| --- | --- | --- | --- | --- | --- | --- | --- | --- | --- | --- |
|  | ICC  [95% CI] | SEM | 95% LOA | | | ICC  [95% CI] | SEM | 95% LOA | | |
|  |  |  | Bias | Lwr | Upr |  |  | Bias | Lwr | Upr |
| **Trunk IMU** | | | | | | | | | | |
| Flexion + Extension | **0.93**  [0.84-0.96] | 7.31 | 4.4 | −14.1 | 22.9 | **0.91**  [0.84-0.95] | 8.17 | −2.8 | −24.1 | 18.6 |
| Lateral flexion L+R | **0.95**  [0.91-0.98] | 4.28 | 2.5 | −17.9 | 23.0 | **0.92**  [0.85-0.96] | 5.04 | −2.8 | −22.7 | 17.0 |
| Trunk-ASMI (Amb) | **0.92**  [0.86-0.96] | 0.64 | −0.2 | −1.9 | 1.6 | 0.88  [0.78-0.93] | 0.72 | 0.2 | −2.0 | 2.3 |
| **Lumbar region IMU** | | | | | | | | | | |
| Flexion + Extension | **0.90**  [0.82-0.95] | 8.37 | 3.4 | −18.1 | 24.9 | 0.87  [0.76-0.93] | 9.46 | 1.1 | −25.7 | 27.8 |
| Lateral flexion L+R | **0.98**  [0.96-0.99] | 2.92 | 0.6 | −7.5 | 8.7 | **0.96**  [0.93-0.98] | 3.84 | −1.0 | −11.3 | 9.3 |
| Lumbar-ASMI (Amb) | **0.94**  [0.89-0.97] | 0.69 | −0.3 | −2.2 | 1.5 | **0.92**  [0.85-0.96] | 0.85 | 0.2 | −2.1 | 2.5 |
|  | | | | | | | | | | |

*n* = 39. All ICC results were statistically significant, *p* < 0.001. Bold denotes ICC >0.9. Abbreviations—ICC: Intraclass correlation coefficient; SEM: standard error of measurement (deg); 95% LOA: 95% limits of agreements (deg)
